# Impact of 2022 flood on socio-economic and health status of people residing in flood-stricken areas of Pakistan: a cross-sectional survey

**DOI:** 10.1097/MS9.0000000000002402

**Published:** 2024-08-02

**Authors:** Qaisar Ali Khan, Ayiz Jan, Sumaira Iram, Iqbal Haider, Aliena Badshah, Arooba Khan, Aabdar Hidayat, Ameer Mustafa Farrukh, Hoor Ul Ain, Ravina Verma

**Affiliations:** a Khyber Teaching Hospital MTI KTH; b Jinnah Medical College, Peshawar; c Saidu Medical College, Swat, PAK; d Sultan Qaboos University, Muscat, Oman; e University of Galway, School of Medicine, Ireland; f St.Georges University, School of Medicine, True Blue, Grenada

**Keywords:** flood, natural disasters, socio-economic status, healthcare, diseases, Pakistan

## Abstract

**Background:**

The events of extreme weather and climate-related disasters such as drought, flood, and heat waves are increasing worldwide. This paper highlights the impact of the 2022 flood in Pakistan on the socio-economic and health status of people residing in flood-stricken areas of Pakistan.

**Methodology:**

A post-flood survey was conducted from three districts of Pakistan with a myriad of questions inquiring about the biopsychosocial aspects of the affected community. Data were collected through a validated questionnaire and was analyzed through SPSS software version 25.

**Results:**

Forty percent of people became homeless; the number of individuals with a low income decreased by 9%; and the number of individuals with a moderate income decreased by 22%. Additionally, 48.7% of subjects lost their income due to flooding, 83.4% of subjects reported having some type of illness or disability since the flooding (previously 16.4%), and 92.8% believed that the floods had affected the health and sanitation of their area. Importantly, 22.6% of subjects expressed difficulty in accessing food after flooding compared to before and 59.9% of respondents noted they were unaware of proper pre-flood evacuation protocols in their area.

**Conclusion:**

The authors’ study indicates that the floods had a significant impact on the socio-economic and health status of residents, particularly those with lower incomes depriving a large proportion of nutrition, health facilities, and property. Without access to necessities and healthcare, individuals are more vulnerable to illnesses, such as chronic respiratory disease, diabetes, and heart disease. The authors stress the importance of providing infrastructural, nutritional, and medical services support to ensure that a community such as Pakistan can recover and thrive post-environmental disaster.

## Introduction

Highlights

- The study highlights the devastating impact of the 2022 flood on the socio-economic and health status of people residing in the flood-affected areas of Pakistan.
- A significant portion of the population became homeless and lacked access to basic food and healthcare facilities.
- The study further found that lack of planning causes more harm and losses, a higher chance of fatalities and injuries, lack of access to medical care, and inadequate disaster response systems, all of which can have a lasting effect on the quality of life in impacted areas.

The destruction of anthropological and ecological systems is greatly attributed to floods. Floods can cause extensive damage to buildings, destroy crops and food sources, contaminate water supplies, and cause the displacement of people and animals. They also have a significant impact on the ecology of an area, altering the landscape and causing species to go extinct. As a result, socio-economic conditions are negatively impacted, public health deteriorates, unemployment is generated, the ecosystem is damaged and severe human casualties occur^1^. As one of the world’s most devastating natural disasters, it is considered one of the most serious. It accounts for 44% of all disaster events from 2000 to 2019^2,3^. The severity and duration of floods are increasing year by year, negatively impacting social and economic conditions in the affected countries. Over the past 20 years, floods have increased in Pakistan at an unprecedented rate, making it one of the most vulnerable countries to water-related disasters. A total of 54 floods of varying intensity have hit Pakistan, ranking it 10th on the Global Environment Risk Index^4,5^. In addition, climate change, such as rising temperatures, melting glaciers in the Himalayas, and variability in monsoons, has made the situation deteriorate^6^. It was the flood that prompted Pakistan’s government to declare a national emergency because of the destruction and casualties caused by it^7^.

As an agricultural country, Pakistan has a vast and well-planned river/ canal system, but the system has been neglected for a long time, resulting in floods for millions of people living in the catchment areas of these rivers and canals. Several major rivers, including the Swat and the Kabul, and several tributaries flow through Khyber Pakhtunkhwa (KP), making flooding a regular risk. Khyber Pakhtunkhwa province was the most severely affected by floods in 2010^8^. In 2022, seventeen districts of the province were adversely affected, leaving 306 people dead^9^. Swat, Dir-Upper, Charsadda, Nowshehra, Tank, and D.I. Khan are among the six most affected districts. The main floods in these areas are caused by low depression monsoon rainfalls that form in the Bay of Bengal. These rainfalls spread across India and Pakistan into the western part of the country. The key factor in flooding was the high intensity of rainfall due to certain known climatic changes in this region. However, considering this crisis, it is crucial to give attention to flood risk management as well^10^. Several rivers and drains flow through Swat, Dir, and Charsadda districts often experiencing floods during the rainy season causing tremendous losses. Charsadda is always endangered by flood risks because the drain flows from north to south in the eastern extremities of the respective districts. There are two rivers in this area: the Jindi River and the Khayali River. Thus this district is prone to both flash floods as well as riverine floods^11^. Another major tributary of the Indus Basin River system is the Swat River. This river is located between the foothills of the Hindukush mountain range also known for its snowcapped peaks. The contribution of snowmelt, average groundwater, and average rainfall in the basin is 65%, 19%, and 16%, respectively, further accounting for the heavy floods in this area^12^.

The social economic impacts are experienced by different people in different ways. This article aims to assess the impact of the 2022 floods on the socio-economic status of residents and their current health status in three different Districts of KPK namely Charsadda, Swat, and Dir.

## Study area

**Figure 1:**
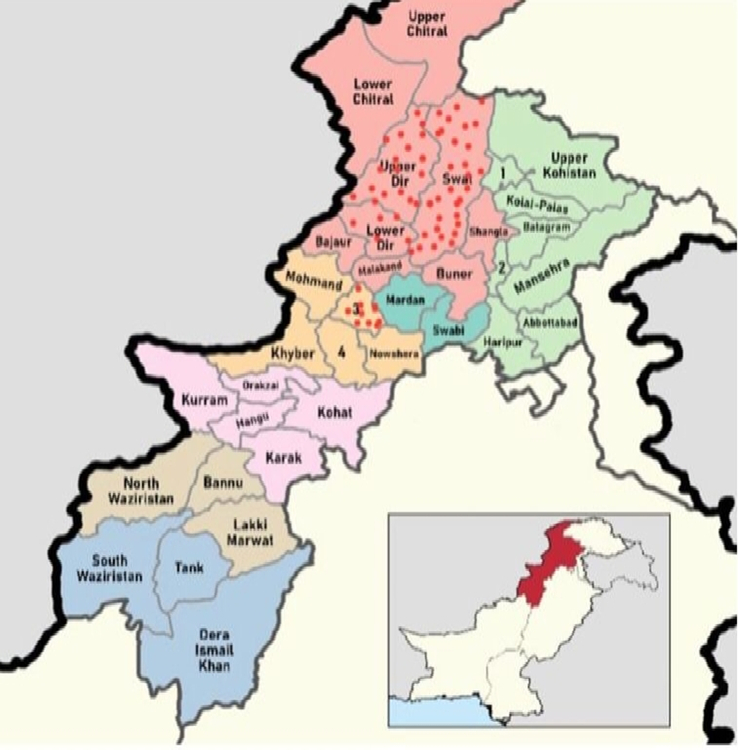
Dots represent the flood-stricken areas of Pakistan; Swat, Dir, and Charsadda^16^.

The research was carried out in three flood-affected districts of Pakistan’s Khyber Pakhtunkhwa (KP) province: Swat, Dir, and Charsadda (Fig. 1).

District Charsadda in KP province of Pakistan. It is located at 34°6'54N 71°41'51E and has an altitude of 273 meters (898 feet). The district is bounded by Mardan in the north, Mohmand agency in the west, and Nowshera in the south. The area is among one of the most fertile regions having mineral-rich soil. It is traversed and bounded by two rivers and two tributaries called as Khialey and Jinday rivers. The average rainfall differs from 300 to 625 mm^13^.

Swat’s total area is 5337 square kilometers, 34°′ to 35° N latitude and 72′ to 74°E′ longitude. In terms of administrative divisions, Swat is surrounded by Chitral, Upper Dir, and Lower Dir to the west, Gilgit-Baltistan to the north, and Kohistan, Buner, and Shangla to the east and southeast, respectively. The Valley of Swat is delineated by natural geographic boundaries and is centered on the Swat River, whose headwaters arise in the 18 000–19 000-foot tall Hindu Kush and Indus River gorges to the east. The average annual rainfall in this region is more than 1000 mm^14^.

Dir is a region in northwestern Pakistan in the Khyber Pakhtunkhwa, in the foothills of the Himalayas. Most of the state lay in the valley of the Panjkora River, which originates in the Hindu Kush mountains and joins the Swat River near Chakdara. Apart from small areas in the south-west, Dir is a rugged, mountainous zone with peaks rising to 5000 m (16 000 ft) in the north-east and to 3000 m (9800 ft) along the watersheds, with Swat to the east and Afghanistan and Chitral to the west and north. Dir typically receives about 127.01 ml (5.0 inches) of precipitation and has 140.36 rainy days (38.45% of the time) annually^15^.

## Materials and methods

### Study design

This cross-sectional survey was conducted from November 2022 to March 2023.

### Sample size

The WHO sample size calculator estimated the sample size by setting the confidence level at 95%, the margin of error at 5%, and the population proportion. The minimum recommended sample size we calculated was 385.

### Sampling strategy

A multi-stage sampling approach was employed to select participants from the flood-affected areas. In the first stage, affected regions were stratified based on geographic location and population density. In the second stage, villages or neighborhoods within each stratum were randomly selected. Finally, households within the selected villages or neighborhoods were approached for participation.

### Data collection and management

A structured questionnaire was formulated and distributed in printed form among the respondents. The questionnaire was written in English, Urdu, and Pushto languages for a better understanding of the local population. Data were collected from both adult male and female populations but due to the cultural restrictions the participation of females was low as compared to males. The questionnaire consisted of four sections that is informed consent, participants’ demographic data, socio-economic, and health status before and after the flood. The questions were closed-ended to ensure the inclusion of important points and the ease of responding to the questions. Door-to-door surveying was conducted for this research because the internet was disconnected in these areas due to flooding. Random sampling from different rural and urban areas of these three districts was done and the participation of the respondents was voluntary. The privacy of the respondents was maintained, and access to the repository data was only given to the core members of the team. The work has been done following the Strengthening the Reporting of Cohort, cross-sectional, and case-control Studies in Surgery (STROCSS) guidelines ^16^.

### Inclusion and exclusion criteria

Participants who had given consent for participation and were above the age of 18 years were interviewed. No restrictions of gender, occupation, and education were applied for conducting this study. Those who are visitors or immigrants from other regions of the country were excluded from the study. Children and those with a lack of decision-making capacity were also excluded from the study.

### Statistical analysis

Descriptive statistics, including means and standard deviations for continuous variables and frequencies and percentages for categorical variables, were calculated to summarize the demographic characteristics of the respondents. χ^2^ tests or Fisher’s exact tests were used to examine associations between demographic variables (e.g. age group, gender, education level) and flood impact indicators (e.g. homelessness, income changes). All the data in the hard copies were transferred to a Microsoft Excel sheet and were analyzed by SPSS version 25 for Windows.

## Results

Among all the 811 participants 696 (85.8%) were males while 113 (13.9%) were females. Two subjects did not disclose their genders. 284 (35.5%) of respondents fall in the age group of 30–40 years, followed by 273 (33.7%) in the age group 40–50 years, 93 (11.5%) came to the age group 50–60 years, 35 (4.3%) were above 60 years of age and 126 (15.5%) were aged below 30. Geographically, 167 (20.6%) participants belonged to District Charsadda, 166 (20.5%) belonged to District Dir, and 478 (58.9%) belonged to District Swat. Regarding occupation of the participants, 135 (16.6%) were running their own business, 79 (9.7%) were in the education sector, 23 (2.8%) were government servants, 21 (2.6%) were healthcare workers, 516 (63.6%) working as laborers, and 37 (4.6%) did not share information about their occupation. The details of the participant’s demographic information are given in Table 1.

**Table 1:** Demographic information of the participants.

Variable	Number (percentage), *n* (%)
Gender	
Female	113 (13.9)
Male	696 (85.8)
NA	2 (0.2)
Age	
30–40	284 (35.0)
40–50	273 (33.7)
50–60	93 (11.5)
Above 60	35 (4.3)
Below 30	126 (15.5)
Location	
Charsadda	167 (20.6)
Dir	166 (20.5)
Swat	478 (58.9)
Education	
None	208 (25.6)
Primary	219 (27.0)
Under-graduate	64 (7.9)
Diploma holder	224 (27.6)
Postgraduate	96 (11.8)
Occupation	
Business owners	135 (16.6)
Educationists	79 (9.7)
Government servants	23 (2.8)
Healthcare workers	21 (2.6)
Labors	516 (63.6)
Unemployed	37 (4.6)
Family size	
12 or more	401 (49.4)
2–4	10 (1.2)
4–8	135 (16.6)
8–12	265 (32.7)
Earning members	
1–2	174 (21.5)
2–4	305 (37.6)
4–6	206 (25.4)
6 or more	76 (9.4)
None	50 (6.2)
Dependent members	
1–2	34 (4.2)
2–4	81 (10.0)
4–6	149 (18.4)
6 or more	532 (65.6)
None	15 (1.8)

NA, not applicable.

### Impact on socio-economic status

**Figure 2:**
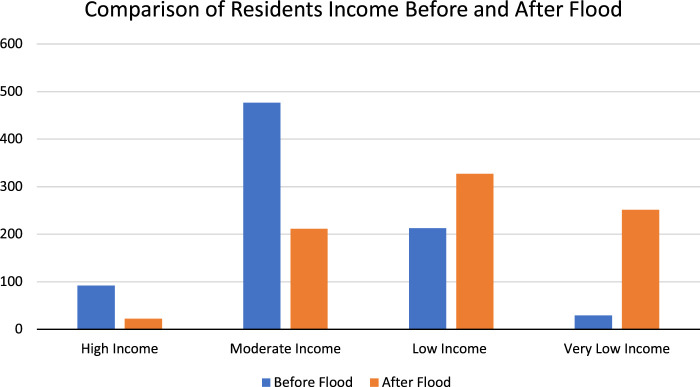
Income distribution before and after the flood.

Socio-economic status (SES) was quantified by employing a multidimensional approach that encompassed several key indicators including participants’ household income, educational attainment, recent occupation, employment status, housing conditions, and asset ownership. Specifically focusing on the impact on participants’ monthly income, a statistically significant difference (*p* value = 0.03) was observed between income levels before and after the flood event. Detailed findings are presented in Table 2, illustrating the changes in income distribution, and further visually depicted in Fig. 2, which graphically represents the pre- and post-flood income distributions.

**Table 2:** Comparison of the monthly income of participants before and after the flood.

	Before flood	After flood	
Current income status	Frequency (percentage), *n* (%)	Frequency (percentage), *n* (%)	*P*
High	92 (11.3)	22 (2.7)	
Moderate	477 (58.8)	211 (26.0)	
Low	213 (26.3)	327 (40.3)	
Very low	29 (3.6)	251 (30.0)	0.03

In surveying participants’ financial standings, 48.7% (395) of subjects lost their source of income due to flood. A statistical difference (*p* value= 0.041) was noted in the employment status of the participants with 402 (49.6%) participants losing their jobs after the flood. In terms of losing jobs, District Swat was most commonly affected and 206 participants lost their jobs, followed by District Dir and Charsaddah (*p* value=0.025). 40 (4.9%) participants became homeless and lost their residential property whereas 263 (32.45%) were shifted to reside in tents. Geographically no statistical difference (*p* value=0.07) was found between the three districts in terms of homelessness.

### Impact on health status

After the floods, 676 (83.4%) were reported to have suffered from various diseases. 753 (92.8%) of subjects believed that the floods had affected the health and sanitation of their respective areas, 98 (12.1%) reported their nutritional status as excellent, 195 (24%) classified nutritional status as fair, 497 (60.7%) classified it as good, 10 (1.2%) said it was poor and 16 (2%) did not answer the question. 183 (22.6%) subjects expressed difficulty in accessing food after the recent floods Regarding the health status, 40 (4.9%) subjects were said to be in an excellent state of health, 252 (31.2%) subjects were in a fair state of health, 478 (58.9%) were in a good state of health, and 41 (5.1%) in a poor state of health. 56 (6.9%) lost their access to a nearby medical facility due to damage to roads, hospital buildings, or transportation due to the flood.

After the flood, respondents commonly suffered from diseases such as diarrhea, malaria, cholera, and hepatitis as given in Table 3. Additionally, Table 4 provides a detailed distribution of these health problems among the respondents based on their age. Regarding pre-flood awareness, 486 (59.9%) subjects were not aware of any pre-flood evacuation protocols, 123 (15.2%) were aware of evacuation protocols, 138 (17%) were unsure of any evacuation protocols and 63 (7.8%) reported that no pre-flood evacuation protocols even exist in their area.

**Table 3:** Distribution of respondents based on reported diseases in flood-affected areas.

Disease	Frequency (percent), *n* (%)
Diarrhea	288 (42.6)
Malaria	201 (29.7)
Cholera	74 (10)
Scabies	22 (3.2)
Hepatitis	20 (2.9)
Enteric fever	13 (1.9)

**Table 4:** Distribution of health problems based on respondents’ age.

	Current health problems, *n* (%)		
Age (years)	Yes	No	Total
Below 30	0	126 (100)	126 (15.5)
30–40	0	284 (100)	284 (35.5)
40–50	5 (1.8)	268 (98.1)	273 (33.7)
50–60	11 (11.8)	82 (88)	93 (11.5)
Above 60	25 (71.4)	10 (28.5)	35 (4.3)
Total	41 (5.1)	770 (94.4)	811 (100)

## Discussion

Floods are the leading cause of death worldwide among climatic disasters. Since the 1950s, floods have increased in frequency and severity due to rising temperatures. Environmental scientists believe that increasing greenhouse gas emissions could be the cause of rising temperatures. Floods are one of the worst natural calamities that can affect the socio-economic development and healthcare services of a nation in the aftermath. Pakistan, a swiftly rising country, has suffered significant impacts from recent severe floods, worsened by population growth and human activities. Major floods devastate the lands of Pakistan approximately every three years, resulting in severe consequences^17,18^.

Pakistan, with the highest number of glaciers outside the Arctic Circle, faces significant vulnerability to floods and other water-related disasters. Because of its agricultural nature, Pakistan has one of the best-planned river and canal systems in the world; the negligence in their maintenance frequently exposes millions of people living in the catchment areas to flood risks. Agricultural production and livestock are particularly susceptible to the risks of climate change, especially in the monsoon season. As a result of the presence of major rivers such as the Swat and Kabul, as well as several tributaries in the Pakistani region of Khyber Pakhtunkhwa (KP), flooding is a frequent risk^19^. Charsadda district administration officials report that high floods in the rivers Swat and Kabul have flooded low-lying areas in the district, affecting thousands of residents^20^.

According to Cutter *et al*.^21^, the social structure of a society has an impact on flood risk. In addition to this, disasters have a significant impact on the political, social, cultural, physical, and economic domains, according to a different study. There is a greater likelihood of damage occurring in economically and socially poor areas, as well as those with less developed infrastructure and lower incomes. In addition to destroying essential infrastructure, natural disasters like floods can cause tremendous damage. In most cases, necessities and facilities, like housing, healthcare, education, water supply, energy, irrigation, and others, are either completely or partially damaged^22^.

Our study reveals that in these agricultural areas of Charsaddah, Swat, and Dir, the impact of floods has encompassed land inundation, erosion, and significant crop losses. There is notable direct damage to critical infrastructure such as houses, roads, and bridges, with the agricultural sector bearing the brunt of these losses. The majority of the population in these areas is made up of laborers or farmers. For small-scale farmers, recovering from these losses is challenging and may necessitate transitioning from on-farm to off-farm work for their livelihoods. Moreover, the floods have also resulted in significant non-economic losses and damages that are difficult to quantify purely in economic terms. These non-economic impacts, such as displacement, emotional distress, and disruption of community life, are particularly profound in developing countries like Pakistan.

Our analysis is consistent with several other studies that have documented substantial damage to infrastructure in flood-affected regions. The recent catastrophic floods in Swat provide a poignant example of these impacts. For the residents of Drab Shahgram village, the 2022 floods marked the second major disaster in just over a decade, following their devastating losses in the July 2010 floods, which destroyed homes, crops, and orchards. In the aftermath of the 2022 disaster, more than 326 897 homes across the region were damaged by floods and landslides, underscoring the widespread destruction. Additionally, the floods led to the tragic loss of 7742 cattle due to collapsing sheds, further exacerbating the humanitarian and economic toll on the affected communities^23,24^. It was reported that, as a result of the destruction of roads and bridges, sending assistance to the affected regions was impossible. In response to the collapse of 495 200 housing areas, ~184 000 Pakistanis were forced to relocate to relief camps with poor sanitation conditions^25^.

Similarly, during the 2010 floods in Charsadda and Swat, a study conducted by Saif and colleagues reported that the schooling of the children was significantly impacted. Floods damaged the boundary walls of houses in the communities, as well as school buildings, classrooms, and furniture. In almost all regions of Pakistan, the floods of 2022 have caused unprecedented destruction and have exceeded the floods of 2010^26^. Inflation touched 27.3% in August due to shrinking foreign exchange reserves. This wide-ranging damage has placed Pakistan in an economic crisis with a loss of around ten billion dollars^27^.

Infrastructure and services must be provided to affected communities to enable them to receive support and assistance. Building capacity and developing systems that enable communities to respond to and recover from disasters is achieved by working with local authorities, NGOs, and other stakeholders. Furthermore, it is important to ensure that affected communities have access to the resources they need to rebuild^28^. As well as ensuring secure places for people to stay and rebuild their homes, flood victims should be provided with food, water, and medical supplies. Additionally, psychological support is essential to helping people cope with the trauma caused by these tragedies^29^.

In terms of efficacy and efficiency, floods pose a great challenge to the healthcare system. By infiltrating aquifers, it can damage access to drinkable water and increase the spread of water-borne diseases. It is possible to categorize health concerns as direct or indirect. In the event of deep water penetration, there may be direct effects, such as death, debris injuries, pollution, hypothermia, and debris injuries. The indirect effects of water disruption include communicable diseases, obesity, famine-related diseases, and diseases related to displaced populations^30^.

In flood-affected areas, gastric issues often arise due to the consumption of contaminated flood water, highlighting the critical role of clean drinking water availability in mitigating health risks. The massive floods ensuing widespread destruction in Pakistan have spawned a serious outbreak of water-borne diseases, which have been discussed by the WHO. According to the study, the most serious morbidity in areas prone to flooding is diarrheal illness. The number of diarrhea cases and malaria cases reported in camps with poor water and sanitation has reached 134 000 and 44 832, respectively. Additionally, skin diseases and eye infections are increasing in prevalence. In flood-stricken areas, 101 snake bites have also been reported^31,32^. As evident from our study, the most common diseases reported among respondents in flood-affected areas were diarrhea, affecting 288 individuals (42.6%), followed by malaria with 201 cases (29.7%), and cholera reported in 74 cases (10%). These diseases reflect the heightened risk of water-borne and vector-borne illnesses in environments where sanitation and clean water access are compromised. In contrast, scabies was the least frequently reported condition, affecting 22 respondents (3.2%), followed by hepatitis and typhoid with 20 (2.9%) and 13 cases (1.9%), respectively.

Similar to our findings, Saif-Ur-Rehman and Shaukat reported that diarrhea emerges as a prevalent gastric problem during floods, affecting 68.4% of respondents. Conversely, about one-third of respondents who avoided using or drinking flood water managed to avoid such gastric issues. Specifically, cholera was identified as a widespread problem, affecting 60% of respondents who used contaminated water, while 40% remained unaffected^33^. Howard, Brillman, and Burkle’s study underscores similar findings, noting increased rates of diarrhea (including dysentery and cholera), hepatitis A and E, respiratory infections, leptospirosis, typhoid fever, and insect-borne diseases in flood-affected developing regions^34^.

Notably, the prevalence of health problems increases with age. Analysis of current health status showed that below 30 years, none of the respondents reported health issues, while in the 30–40 age group, no health problems were reported among individuals. However, the frequency of health problems rises sharply with age; among respondents aged 50–60 years, 11 individuals (11.8%) reported health problems, and in the above-60 years category, 25 individuals (71.4%) reported health issues. A clear relationship has been observed between the age of respondents and their physical health condition, indicating that vulnerability to health problems increases with age^35,36^. This necessitates continuous health monitoring and care for elderly individuals, who are more susceptible to health challenges due to factors such as reduced immunity associated with aging. Addressing the healthcare needs of older populations becomes increasingly critical to mitigating the impact of age-related health vulnerabilities.

Lack of governmental support and resources further exacerbates the situation, leading to a vicious cycle of poverty and homelessness. Further, individuals living in poverty often lack access to healthy food, which results in a high rate of obesity and malnutrition, which further contribute to chronic illnesses. The decrease in nutritional status is likely due to the loss of access to food sources as well as a lack of access to necessities. In these circumstances, it is even more difficult for the community to recover and rebuild^37^. To our surprise, the nutritional analysis revealed that a significant majority of respondents reported maintaining good nutritional status. This was largely attributed to their relocation to areas less affected by flooding, where roads remained accessible, and where community efforts ensured the availability of essential food supplies. The strategic move to safer locations allowed individuals to continue accessing nutritious food, thereby mitigating the potential impact of the floods on their nutritional wellbeing.

Conversely, it is crucial to address the needs of individuals who lack access to such facilities. It is recommended that the government of Pakistan prioritize this issue, especially given the current circumstances, by ensuring accommodation for displaced victims and providing essential food supplies. In cooperation with the government, non-governmental organizations need to provide victims with necessities, such as clean drinking water to prevent the spread of water-borne diseases, medications to treat those who have already been afflicted, and feminine hygiene products for women.

The study is limited by a lack of sufficient information from females regarding access to food, medical care, and current health conditions. Because of the randomized sampling used in the study, a significant proportion of severely affected communities may not be included, as well as floods that severely damaged roads and made isolated areas inaccessible. The data collection is likely to have been insufficient because a large number of people migrated to other safe locations before the survey was conducted. Moreover, the nutrition status of respondents was not adequately assessed in this study, and further study is needed.

## Conclusion

This study indicated that the floods significantly affected the population, particularly those with lower incomes. A significant portion of the population became homeless and lacked access to basic food and healthcare. We further found that lack of planning causes more harm and losses, a higher chance of fatalities and injuries, lack of access to medical care, and inadequate disaster response systems, all of which can have a lasting effect on the quality of life in impacted areas. With a considerably large number of people residing in locations vulnerable to flooding and other environmental catastrophes, it becomes difficult for a community like Pakistan to recover and flourish after an environmental disaster, therefore, it is imperative to encourage the development of infrastructure, nutritional reform, and healthcare to ensure quick recovery and to return to a state of stability and progress.

## Ethical approval

The ethical review approval was granted by the Institutional Review Ethical Board Khyber Teaching Hospital Peshawar Pakistan with reference number 74/DME/KMC dated 10 February 2023

## Consent

Written informed consent was obtained from the patient for publication of this study and accompanying images. A copy of the written consent is available for review by the Editor-in-Chief of this journal on request.

## Source of funding

No funding sources were received for conducting this study of this research.

## Author contribution

Q.A.K., A.J., I.H. conceptualized the idea. Q.A.K., A.K., A.J. collected the data. A.B., and A.H. Analyzed the data. H.U.A., A.M.F., R.V. and A.J. contributed to manuscript writing. Q.A.K., I.H., and A.B. critically revised the manuscript and did the final editing. All the authors approved the final manuscript before submission

## Conflicts of interest disclosure

The authors report no conflicts of interest.

## Research registration unique identifying number (UIN)

Registration body: Research Registery.

Unique Identification Number (UIN): researchregistry10208

https://researchregistry.knack.com/research-registry#userresearchregistry/registerresearchdetails/6625455941304e0028130827/

## Guarantor

Qaisar Ali Khan.

## Data availability statement

Data can be available on a reasonable request to the corresponding author.

## Provenance and peer review

Not commissioned, externally peer-reviewed.
